# Developing an innovative chimeric multi-epitope subunit vaccine against *Staphylococcus intermedius* using an immunoinformatics strategy via Multi-omics approaches

**DOI:** 10.1515/med-2025-1281

**Published:** 2026-03-18

**Authors:** Muhammad Naveed, Furrmein Fatima, Sarmad Mahmood, Tariq Aziz, Nimra Hanif, Nausheen Nazir, Ashwag Shami, Maher S. Alwethaynani, Fakhria A. Al-Joufi, Bandar K. Baothman, Sarah Almaghrabi, Majid Alhomrani

**Affiliations:** Department of Biotechnology, Faculty of Science and Technology, University of Central Punjab, Lahore, Pakistan; Laboratory of Animal Health, Hygiene and Food Quality, University of Ioannina Arta, 47132, Arta, Greece; Department of Biochemistry, University of Malakand Chakdara, Dir Lower Pakistan; Department of Biology, College of Science, Princess Nourah bint Abdulrahman University, P.O Box 84428, Riyadh, 11671, Saudi Arabia; Department of Clinical Laboratory Sciences, College of Applied Medical Sciences, Shaqra University, Alquwayiyah, Riyadh, Saudi Arabia; Department of Pharmacology, College of Pharmacy, Jouf University, Aljouf, Saudi Arabia; Department of Medical Laboratory Sciences, Faculty of Applied Medical Sciences, King Abdulaziz University, Rabigh, Saudi Arabia; Department of Medical Laboratory Sciences, Faculty of Applied Medical Sciences, King Abdulaziz University, Jeddah, 21589, Saudi Arabia; Department of Clinical Laboratory Sciences, The Faculty of Applied Medical Sciences, Taif University, Taif, Saudi Arabia

**Keywords:** *Staphylococcus intermedius,* pan-genome, immunogenicity, craniofacial osteomyelitis, brain abscesses, vaccine

## Abstract

**Background:**

*Streptococcus intermedius* is a major human pathogen associated with invasive diseases such as meningitis and endocarditis. These infections may lead to inflammation, fever, and cardiac damage. At present, no effective vaccine exists for prevention.

**Objectives:**

This study aimed to design a stable, non-allergenic, and antigenic chimeric multi-epitope vaccine against *S. intermedius* using Immunoinformatics approaches.

**Methods:**

Twelve B-cell, five helper T lymphocyte (HTL), and five cytotoxic T lymphocyte (CTL) epitopes were predicted using advanced immunoinformatics tools. These epitopes were assembled into a single vaccine candidate. The construct was evaluated in silico for its antigenicity, allergenicity, and physicochemical stability. A 3D structural model of the vaccine was generated and validated. Molecular docking and dynamics simulations were conducted to assess interactions between the vaccine and immune receptors: TLR4, TLR3, MHC-I, and MHC-II.

**Results:**

The final vaccine candidate demonstrated favorable antigenic and non-allergenic properties, along with high stability. Structural validation confirmed proper folding. Docking analyses revealed strong binding affinities between the vaccine and target immune receptors. Molecular dynamics simulations indicated stable complexes, supporting the construct’s immunological compatibility.

**Conclusions:**

The designed chimeric multi-epitope vaccine shows strong potential to elicit an immune response against *S. intermedius*. These findings provide a foundation for further experimental validation through *in vivo* and clinical trials.

## Introduction

Streptococcus Intermedius (SI) is a Gram-positive bacterium that belongs to the *Streptococcus anginosus* group [1]. *Streptococcus intermedius* was first identified in the United Kingdom. It is a commensal microorganism of the human oral, gastrointestinal, and genitourinary tracts [2]. However, under specific conditions, it can become pathogenic and cause various infections in humans and associated with a range of infections, including abscesses in deep-seated tissues such as the liver, brain, and abdomen, as well as infections in the oral cavity and respiratory tract [3]. SI becomes pathogenic under conditions of compromised immunity, allowing it to cause infections, including abscesses in deep-seated tissues (liver, brain, abdomen), as well as in the oral cavity and respiratory tract. Its ability to form abscesses and its potential to cause systemic infections are notable pathogenic effects, often requiring appropriate medical intervention and treatment [4]. These infections pose significant challenges due to their high morbidity and mortality rates, as well as the emergence of antibiotic-resistant strains [5].

The pathogenesis of SI involves a complex interplay between the bacterium and the host immune system. One of the key virulence factors in SI is Intermedilysin (ILY), a pore-forming toxin encoded by the intermedilysin gene [6]. Upon colonization of a host’s mucosal surface, SI secretes ILY, which binds to the glycosylphosphatidylinositol-anchored protein human CD59 (hCD59). This interaction triggers a cascade of conformational changes within ILY, leading to the formation of oligomeric membrane-embedded pore complexes [7].

Production of a vaccine for this bacterium poses considerable challenges due to limited knowledge of bacterium pathogenicity as well as the absence of suitable animal models for testing, and the intricate nature of immune interactions [8]. However, contemporary in-silico methodologies offer a compelling avenue with promising potential for the development of vaccines against infectious diseases. These innovative techniques involve simulating and analyzing various interactions of pathogen [9].

The fields of genomics and bioinformatics have contributed to the emergence of novel methodologies for the identification of antigenic vaccine targets within bacteria [10]. The Bacterial Pan Genomic Analysis (BPGA) technique has demonstrated significant potential in the recognition of potential vaccine candidates through the examination of conserved protein families. This method also holds promise for the purpose of vaccine target identification [11].

Pan-genomic analysis includes the comparative analysis of multiple genomes originating from a single species of bacteria. This analytical approach enables the identification of both the core genomic sequences and conserved protein sequences, encompassing genes that maintain a universal presence across all strains of the given species [12]. In the specific case of *S. intermedius*, an inclusive bacterial pan-genomic analysis was undertaken, focusing on the identification of critical features based on accessory components, sequence conservation, and core proteins that shows no similarity to human proteins. The core proteome containing genes potentially involved in virulence or mediating interactions between the host and pathogen emerges as a valuable reservoir of vaccine targets [13].

The proposed vaccine candidate is specifically designed to target various surface exposed proteins and secreted virulence factors, such as lipases, exotoxins, and adhesins, which are crucial in the pathogenic process of *S. intermedius*. This study aims to generate a vigorous immune response and deliver effective defense against *S. intermedius* infections [14]. Utilizing subtractive proteomics and genomics methods, the research focuses on creating an in-silico chimeric vaccine design targeting *S. intermedius*.

## Methodology

### Proteomic data collection and core protein selection

The complete proteome sequence of *Streptococcus intermedius* was obtained from NCBI Genome collection. To identify core proteins within the database, we utilized the Bacteria Pan Genomic Analysis (BPGA) which employs the USEARCH clustering tool by default. This approach facilitated the identification of conserved protein families common among various *Streptococcus intermedius* (Uniprot Taxon ID: 1285) strains. Key data gathered included strain names, isolation sources, and geographic origins [15].

**Ethical approval:** Not applicable.

## Screening of essential virulent core peptides

Core protein families identified from different *Streptococcus intermedius* species were analyzed using BLASTp to gather essential protein information. BLASTp searches were conducted against the Database DEG10 with parameters set at an E-value ≤100ˆ-5 and bit score≥98 to ensure accurate identification of antigenic peptides [16]. Major proteins were further analyzed by VFdb database for the virulence factors and by Microbial Virulence Database (MvirDB) [17]. Resistance-associated proteins were identified through BLASTp searches against the Antibiotic Resistance Gene-Annotation Database and Comprehensive Antibiotic Resistance Database [18].

## Filtering homologous and non-homologous proteins

**Figure 1:**
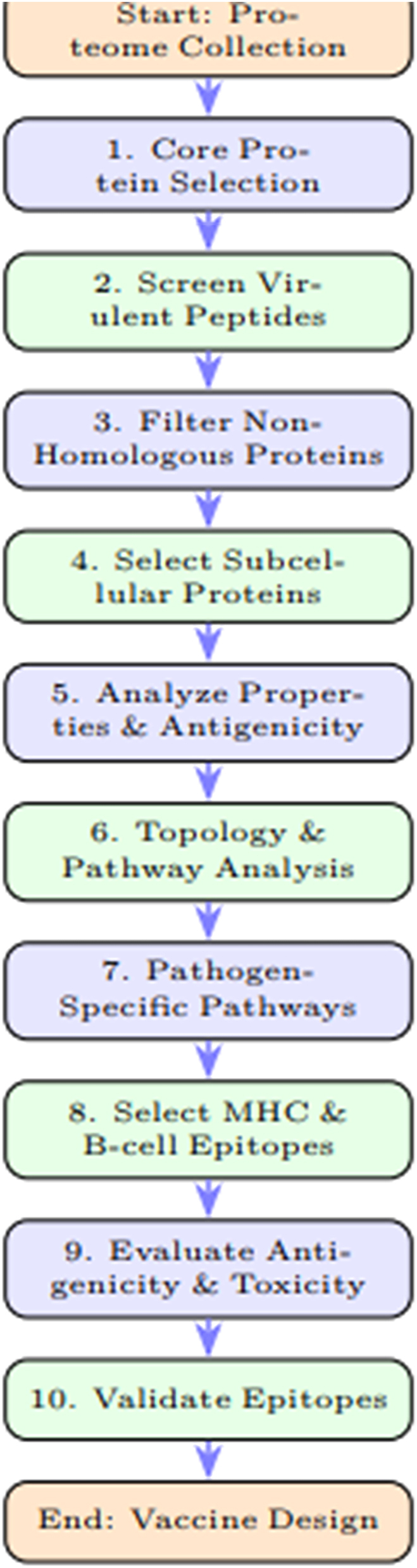
Flowchart of the methodology for designing a chimeric multi-epitope vaccine against *Streptococcus intermedius*.

The necessary resistance-associated and virulent proteins were filtered out from the human proteome using BLASTp [19]. This step aimed to exclude proteins homologous to human gut flora proteins, ensuring that only non-homologous proteins are considered for further analysis (Figure 1).

## Selection of subcellular localized proteins

Subcellular localization of the proteins was predicted using two online servers: CELLO v2.5 and PSORTb v3.0.2. PSORTb determined various subcellular localizations such as outer membrane, cytoplasmic membrane, cytoplasmic, extracellular and periplasm. CELLO was used to further validate these predictions [20].

## Analysis of physiochemical properties and antigenicity

The physicochemical properties of selected proteins were determined using Expasy ProtParam, Antigenicity was determined by VaxiJen and AntigenPRO webserver which assesses antigenicity potential of the proteins based on physicochemical properties [21].

## Protein topology and pathway analysis

Proteins embedded in the plasma membrane and those being exported were analyzed using the TMHMM v.2.0 server, which predicts protein topology using the Markov method [22]. SignalP 5.0 was employed for secretory pathway analysis, using a deep neural network to predict signal peptide sequences and classify them.

## Pathogen-specific pathways and functional analysis

Metabolic pathways of *Streptococcus intermedius* were compared manually with human pathways using the KEGG pathway database to identify unique and shared pathways [23]. Protein function prediction was conducted using UniProt, the KEGG Genes Database, and InterPro servers.

## Selection of MHC-I, MHC-II, and B cell epitopes

MHC Class I and MHC Class II epitope prediction was performed using the Immune Epitope Database (IEDB) server with the Artificial Neural Network (ANN) method based on algorithms of NetMHCpan-4.1 and NetMHCIIpan-4.0, respectively. Protein sequences in FASTA format were analyzed to identify 8–11-mer peptides for MHC Class I and 15-mer peptides for MHC Class II, with a stringent IC50 threshold of <100 nM to select high-affinity binders. Epitope selection prioritized non-allergenicity (via AllerTOP v2.0), high antigenicity (VaxiJen v2.0, threshold >0.5), and non-toxicity (ToxinPred), ensuring immunogenic potential without adverse effects. Peptide-MHC stability was assessed using NetMHCstabpan for MHC Class I, and multi-allele binding was prioritized for MHC Class II to enhance population coverage.

## Evaluation of antigenicity, toxicity, and allergenicity

Antigenic properties of MHC Class II, B-cell epitopes and MHC Class I were determined using VaxiJen v2.0. Toxicity prediction was performed using ToxinPred, which evaluates physicochemical properties to ensure specific targeting of bacterial epitopes without adverse host effects. Allergenicity was analyzed using the AllerTOP v2.0 server [24].

## Validation of epitopes: conservancy, hydrophobicity, and IFN induction

Conservation levels of specific epitopes were assessed using the IEDB web server with a 100 % sequence identity threshold. Hydrophobicity profiles were determined using the ProtParam server, calculating the GRAVY value for a comprehensive analysis. IFN-gamma immune response induction by selected Helper T cell (HTL) epitopes was evaluated using the IFN epitope server, employing motif and support vector machine (SVM) hybrid methods [25].

## Secondary and three-dimensional (3D) epitope structure prediction

To determine the 3D structures of selected MHC Class II and MHC Class I epitopes, the Alphafold two webserver was employed. The sequence was submitted to the server, and it predicted the structure using its neural network-based algorithm that leverages multiple sequence alignments (MSAs) and a structure module. These server-generated *de novo* peptide 3D structures provide visual insights into the spatial arrangement of the epitopes [26]. Additionally, the secondary structures of the vaccine epitopes were predicted using the PSIPRED tool, which provided information on alpha helices, beta sheets, and random coils within the protein structure. AlphaFold2 was selected for its proven reliability, open-source accessibility, and sufficient accuracy in predicting the protein structure, with subsequent refinement steps improving the structure to a quality comparable to AlphaFold3’s output, aligning with our study’s needs, though we plan to utilize AlphaFold3 for future investigations.

## Molecular docking with human receptors

To analyze the binding interactions between the epitopes and major histocompatibility complex (MHC) molecules, molecular docking techniques were utilized [27]. Initially, the PatchDock tool facilitated docking experiments, generating potential complex structures between the epitopes and MHC molecules [28] These models were subsequently refined and re-scored using the FireDock server, which categorized them based on their global energy values, with lower values indicating more favorable binding interactions. Following this, molecular dynamic simulations of the selected epitopes with Toll-like receptors (TLRs) were conducted using the iMODS server.

## Molecular dynamics simulations

Molecular dynamics simulations were performed using the IMODS server. The IMODS server’s robust computing capabilities and specialized configurations made it well-suited for managing the complex computational tasks involved in simulating molecular systems. This enabled researchers to investigate intricate molecular interactions and dynamics with high precision and efficiency, furthering our understanding of molecular phenomena.

## Binding energy calculations

The lowest binding energies of the docked complexes were calculated using the HawkDock server. This evaluation employed MM-PB/GBSA method, allowing for the assessment of both the lowest binding free energies of the vaccine-immune receptor complexes and the other mechanical energies associated with the docked complexes involved in the interaction [29].

## Results

### Bacteria pan-genomic analysis

**Figure 2:**
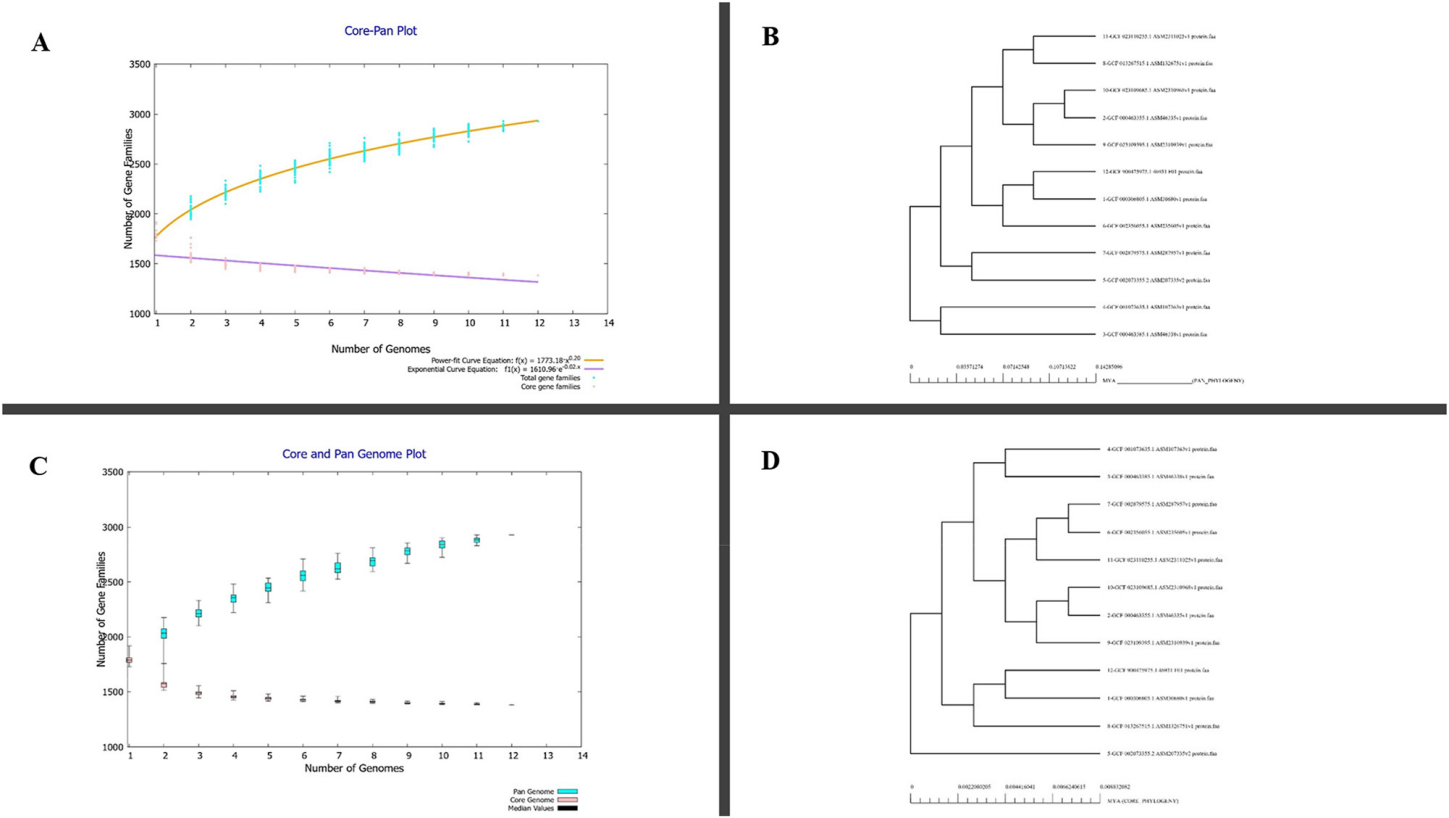
Pangenome Analysis of *Staphylococcus intermedius.* (A) Number of gene families in 12 different strains of *Staphylococcus intermedius* and graphical representation of PAN genome (B) phylogenetic tree of 12 different strains of *Staphylococcus intermedius and* PAN phylogeny (C) graphical representation of CORE proteome (D) graphical representation of core phylogeny.

The core proteomes of 12 Strains of *Streptococcus intermedius* (Uniprot Taxon ID: 1285) were sequenced and it identified almost 41,212 different types of proteins in all strains. The Pan-genome analysis of *streptococcus intermedius* shown in Figure 2, contains the entire collection of genes present in the strains. A tool CDHit server was employed to simplify and filter out the redundancy. This step resulted in the identification of 532 unique proteins, with each protein represented only once (Figure 1). Moreover, the cellular localization of non-redundant proteins was analyzed, which resulted in the identification of 82 membrane proteins, 41 cytoplasmic proteins, nine lipoproteins, and 32 secreted proteins.

## Prediction of B-cell epitopes and physiochemical properties assessment

The selected protein was subjected to epitope prediction and 12 epitopes with a length of 16 amino acids were chosen for use in the vaccine’s final design. Allergenicity and antigenicity were predicted for the selected epitopes showing that all epitopes were non-allergenic and had an antigenicity score greater than 0.5. These were the top vaccination candidates for eliciting an immunological response (Table 1).

**Table 1:** B cell epitopes prediction by IEDB server.

B Cell – epitopes	Allergenicity	Antigenicity
YETIRSRSWSGNGYNR	Non-allergenic	Antigenic (0.74)
TGLAWEPWRLIYSKND	Non-allergenic	Antigenic (0.60
ISTWGTTLHPQFEDKV	Non-allergenic	Antigenic (0.96)
AAFAETPTKPKAAQTE	Non-allergenic	Antigenic (0.51)
GHDADGYETIRSRSWS	Non-allergenic	Antigenic (1.01)
TLHPQFEDKVVKDNTD	Non-allergenic	Antigenic (1.10)
NGESNLTVENPSNSTV	Non-allergenic	Antigenic (1.08)
QSMSQLQAKFGADFSK	Non-allergenic	Antigenic (0.66)
AVPARMQYESISAQSM	Non-allergenic	Antigenic (0.86)
GNPGEASKVITGNIDT	Non-allergenic	Antigenic (0.94)
LNDYIWGLQYDKLNIL	Non-allergenic	Antigenic (0.61)
NSEAAKKALNDYIWGL	Non-allergenic	Antigenic (0.97)

## T-cell epitope prediction

**Figure 3:**
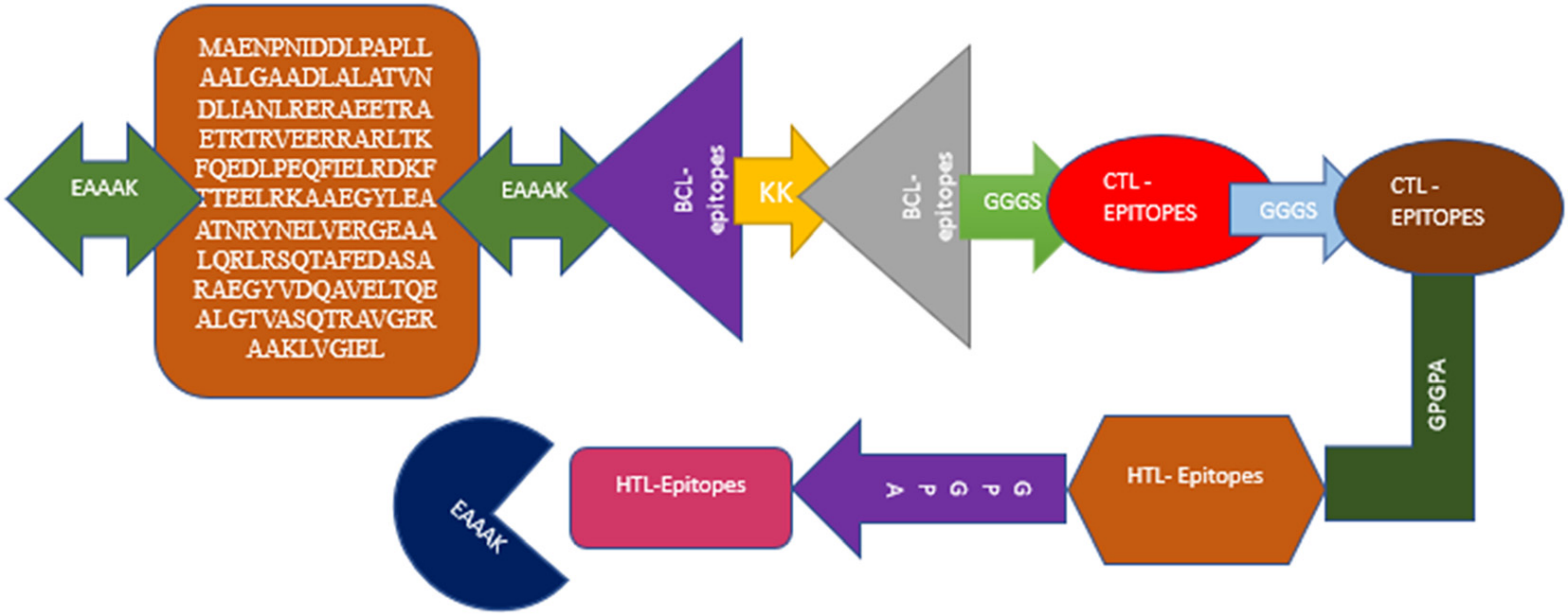
Flow chart design of vaccine candidate.

**Figure 4:**
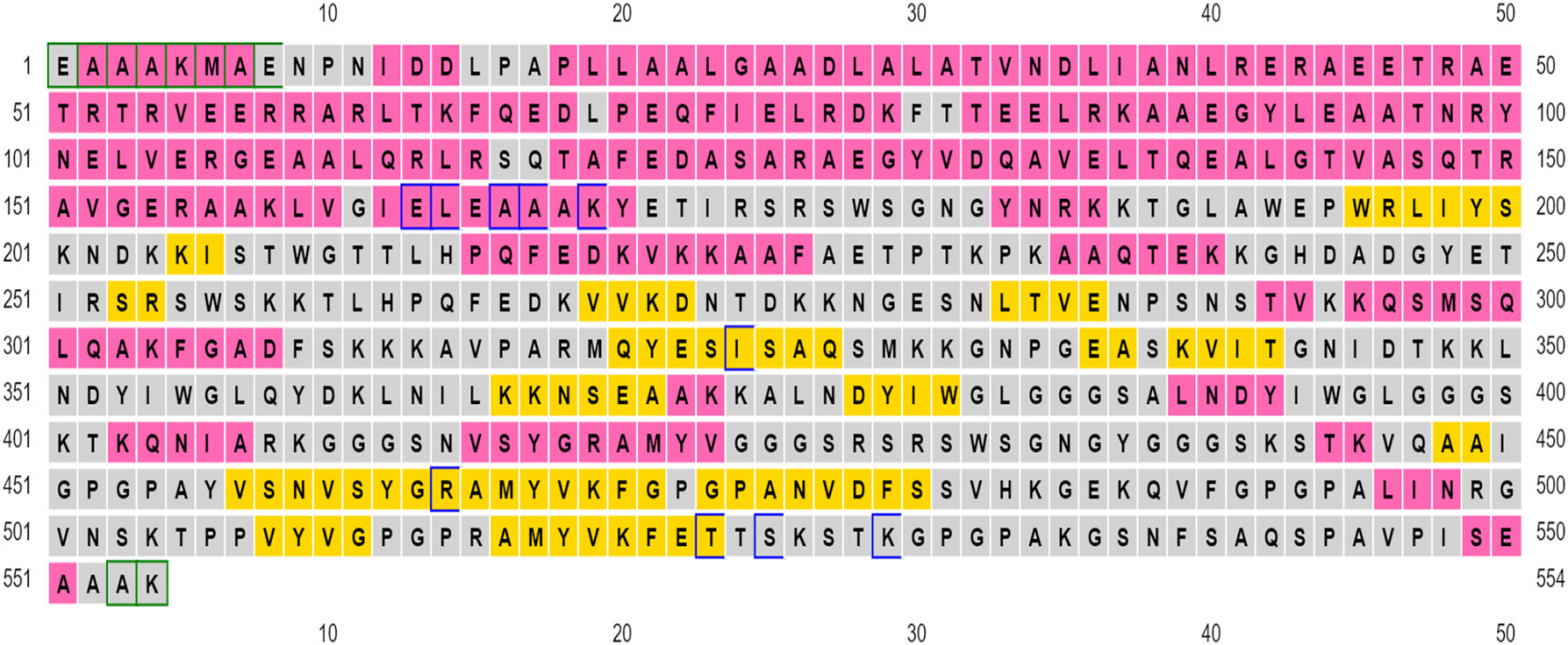
Secondary structure of vaccine construct showing the count of alpha halix (pink), beta sheets (yellow) and coils (grey) in the vaccine construt.

The selected protein’s predicted T-cell epitopes of MHC Class-II and I had a sequence length of 16 and nine amino acids, respectively. These targeted epitopes were then further evaluated based on the highest MHC binding alleles. The lowest percentile scores were utilized to identify MHC-II and MHC-I epitopes that can activate cellular immunity using B-cell epitopes created from T-cell epitopes. Epitopes were selected on the basis of IC50 score lower then 100, non-allergenicity, non-toxicity and antigenicity score higher than 0.5 threshold. analyses were done, predicting that these CTL and HTL epitopes are non-toxic, confirmed by tool ToxinPred and have been used for vaccine development (Table 2 and Table 3). The IEBD population coverage tool was used to analyze the combined population coverage of MHC-I and MHC-II, resulting in a remarkable 87 % global coverage, demonstrating strong potential as a vaccine candidate (Figures 3 and 4).

**Table 2:** MHC I predicted epitopes.

Epitopes	Allergenicity	Antigenicity	Toxicity	Human alleles
ALNDYIWGL	Non-allergenic	Antigenic	Non toxic	HLA-A*02:01 HLA-A*02:03 HLA-A*02:06 HLA-A*32:01 HLA-B*15:01 HLA-A*68:02 HLA-A*31:01 HLA-B*08:01
KTKQNIARK	Non-allergenic	Antigenic	Non toxic	HLA-A*30:01 HLA-A*03:01 HLA-A*11:01 HLA-A*68:01 HLA-A*30:02 HLA-B*57:01 HLA-A*32:01 HLA-B*15:01
NVSYGRAMYV	Non-allergenic	Antigenic	Non toxic	HLA-A*68:02 HLA-A*02:03 HLA-A*02:06 HLA-A*02:01 HLA-A*30:02 HLA-B*57:01 HLA-A*68:01 HLA-A*03:01
RSRSWSGNGY	Non-allergenic	Antigenic	Non toxic	HLA-A*30:02 HLA-B*15:01 HLA-A*30:01 HLA-B*57:01 HLA-A*03:01 HLA-A*01:01 HLA-B*58:01 HLA-A*11:01
KSTKVQAAI	Non-allergenic	Antigenic	Non toxic	HLA-B*58:01 HLA-B*57:01 HLA-A*32:01 HLA-A*30:01 HLA-A*30:02 HLA-A*68:02 HLA-A*02:03 HLA-A*31:01

**Table 3:** Selected MHC II predicted epitopes.

Epitopes	Allergenicity	Antigenicity	Toxicity	Human alleles
YVSNVSYGRAMYVKF	Non-allergenic	Antigenic	Nontoxic	HLA-DRB1*07:01 HLA-DRB1*15:01 HLA-DRB5*01:01 HLA-DRB3*02:02 HLA-DRB3*01:01 HLA-DRB1*03:01 HLA-DRB4*01:01
NVDFSSVHKGEKQVF	Non-allergenic	Antigenic	Non toxic	HLA-DRB5*01:01 HLA-DRB1*07:01 HLA-DRB1*03:01 HLA-DRB1*15:01 HLA-DRB3*02:02 HLA-DRB3*01:01 HLA-DRB4*01:01
LINRGVNSKTPPVYV	Non-allergenic	Antigenic	Nontoxic	HLA-DRB1*07:01 HLA-DRB3*02:02 HLA-DRB5*01:01 HLA-DRB1*15:01 HLA-DRB1*03:01 HLA-DRB3*01:01 HLA-DRB4*01:01
RAMYVKFETTSKSTK	Non-allergenic	Antigenic	Nontoxic	HLA-DRB4*01:01 HLA-DRB5*01:01 HLA-DRB1*15:01 HLA-DRB1*03:01 HLA-DRB1*07:01 HLA-DRB3*01:01 HLA-DRB3*02:02
KGSNFSAQSPAVPIS	Non-allergenic	Antigenic	Non toxic	HLA-DRB1*07:01 HLA-DRB3*02:02 HLA-DRB5*01:01 HLA-DRB4*01:01 HLA-DRB3*01:01 HLA-DRB1*15:01 HLA-DRB1*03:01

## Construction and processing of multi-epitope vaccine

**Figure 5:**
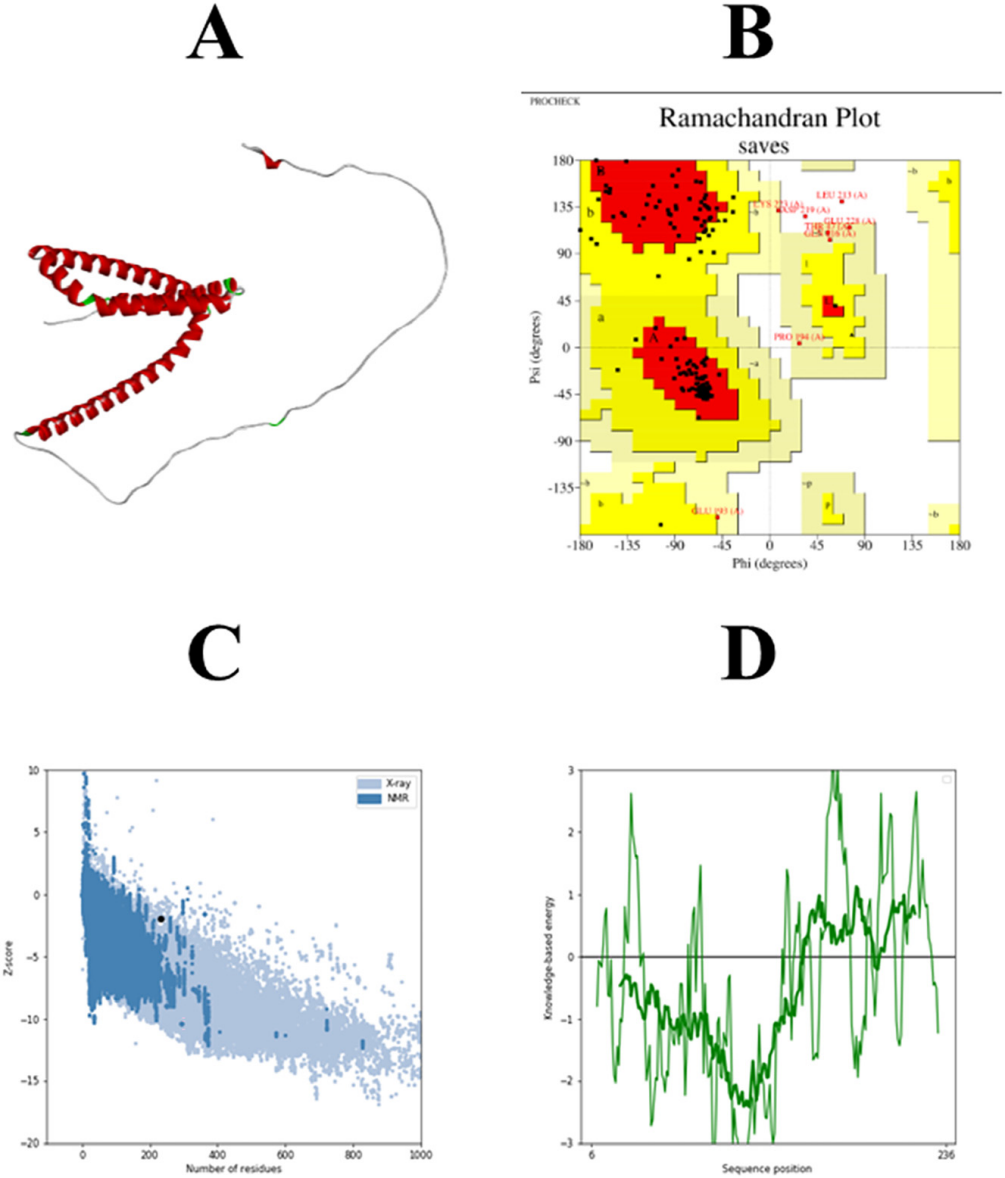
Tertiary structure analysis of vaccine. (A) 3D structure of vaccine predicted by alpha fold2 (B) the ramachandran plot analysis (C) overall model quality predicted Pro-SA web (D) local model quality.

An epitope-based vaccine combines multiple epitopes to enhance immune response effectiveness against different variations of a pathogen. In this study, vaccine candidates were designed by integrating an adjuvant, a PADRE sequence, B cell epitopes (BCL), cytotoxic T lymphocyte epitopes (CTL, MHC-I), and helper T lymphocyte epitopes (HTL, MHC-II). The vaccine candidate includes 12 B cell epitopes, 5 CTL epitopes, and 5 HTL epitopes, using an HBHA conserved sequence as the adjuvant. The design process involved linking these components with specific linkers to ensure optimal assembly and function. The adjuvant sequence was first connected to the PADRE sequence using an EAAAK linker. The PADRE sequence was then linked to the B cell epitopes using a KK linker, and subsequent B cell epitopes were also joined with KK linkers. To connect BCL epitopes with CTL epitopes, a GGGS linker was used, and the CTL epitopes were similarly connected to each other using GGGS linkers. The GPGPA linker facilitated the linkage of CTL epitopes to HTL epitopes, and HTL epitopes were also connected using GPGPA linkers. The final step in constructing the vaccine was to append an EAAAK linker at the end of the sequence to complete the design (Figure 5).

## Prediction of physiochemical properties of vaccine candidate

The vaccine candidate consists of 555 amino acids with a molecular weight of 60,193.59 kDa. The theoretical PI value is 9.62 indicating that it has a negative charge. The instability index value of the vaccine is computed to be 36.91 showing vaccine stability. The aliphatic index of 65.17 represents vaccine stability in different temperatures. The vaccine’s negative GRAVY values demonstrated its hydrophilic nature. The estimated half-life of the vaccine is >10 h in *E. coli* showing its good solubility. Thus, vaccine design was found to be non-allergenic, soluble, hydrophilic, antigenic, and stable.

## Non-homology analysis

The designed vaccine candidate shows no similarity to the human proteome, human gut microbiota, or mouse proteins, as predicted by the PBIT server. Consequently, it is unlikely that the vaccine will induce immunological tolerance or cause unforeseen effects upon administration. Furthermore, the vaccine candidate construct demonstrated a 64 % sequence similarity to *Streptococcus intermedius* in the BLAST analysis, confirming that it has retained the key features necessary for accurately identifying its original proteins and organisms.

## Secondary structure prediction of vaccine construct

**Figure 6:**
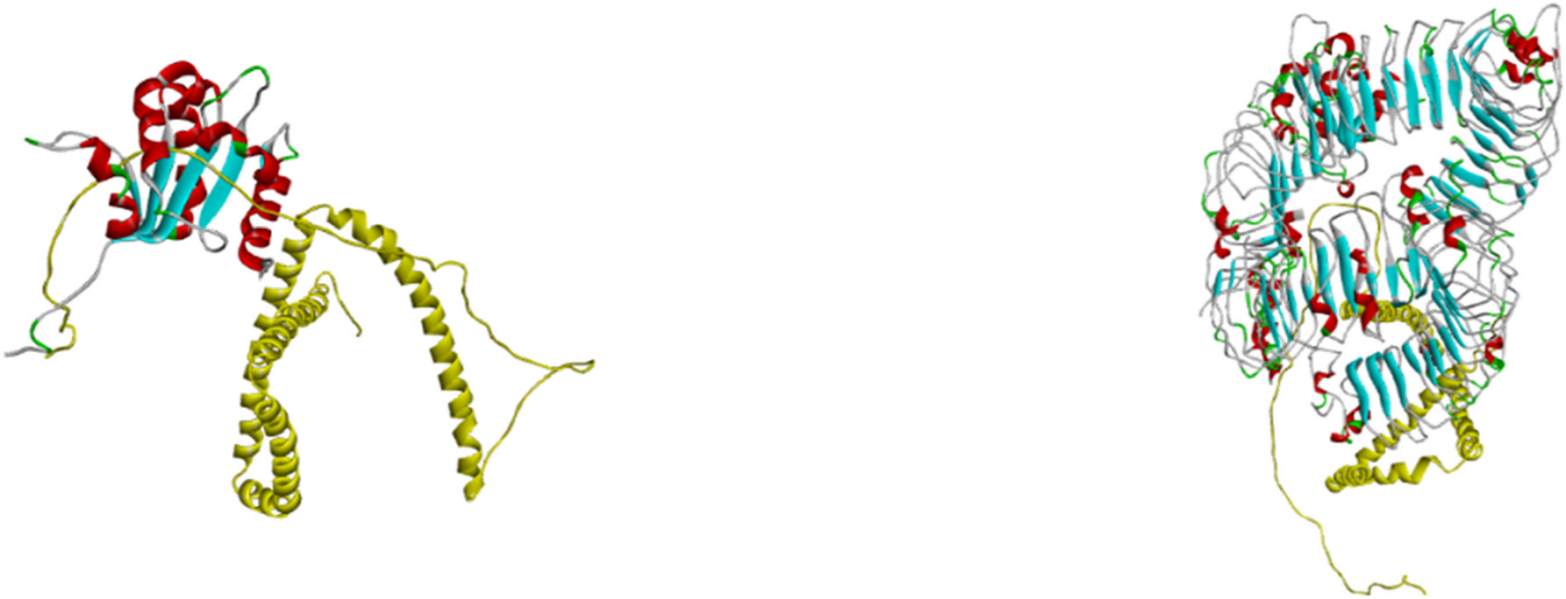
Molecular docking analysis of the designed vaccine with Toll-like receptors. (A) Docked complex of the vaccine with TLR1. (B) Docked complex of the vaccine with TLR5.

The secondary structure of the vaccine candidate was predicted by the PSIPRED webserver. The results demonstrated that designed vaccine contains a-helix, 42.70 % (237 amino acids), b-sheet, 23.47 % (78 amino acids), and random coils, 33.87 % (188 amino acids). The vaccine candidate has a high number of alpha helix and random coils as compared to beta sheets (Figure 6). Moreover, according to MEMSAT analysis the majority of amino acids in the vaccine contruct were also projected to be cytoplasmic, suggesting the probability of cellular penetration. 1–19 amino acid residues, 20–35 amino acid residues, and 36–554 amino acid residues were found to be in the extracellular region, transmembrane helix, and cytoplasmic region, respectively. According to PSIPRED, protein disorder in the vaccine candidate was predicted to be fairly low, with only 3 % of residues being classified as disordered using a threshold value of 0.6.

## Prediction and validation of vaccine tertiary structure

**Figure 7:**
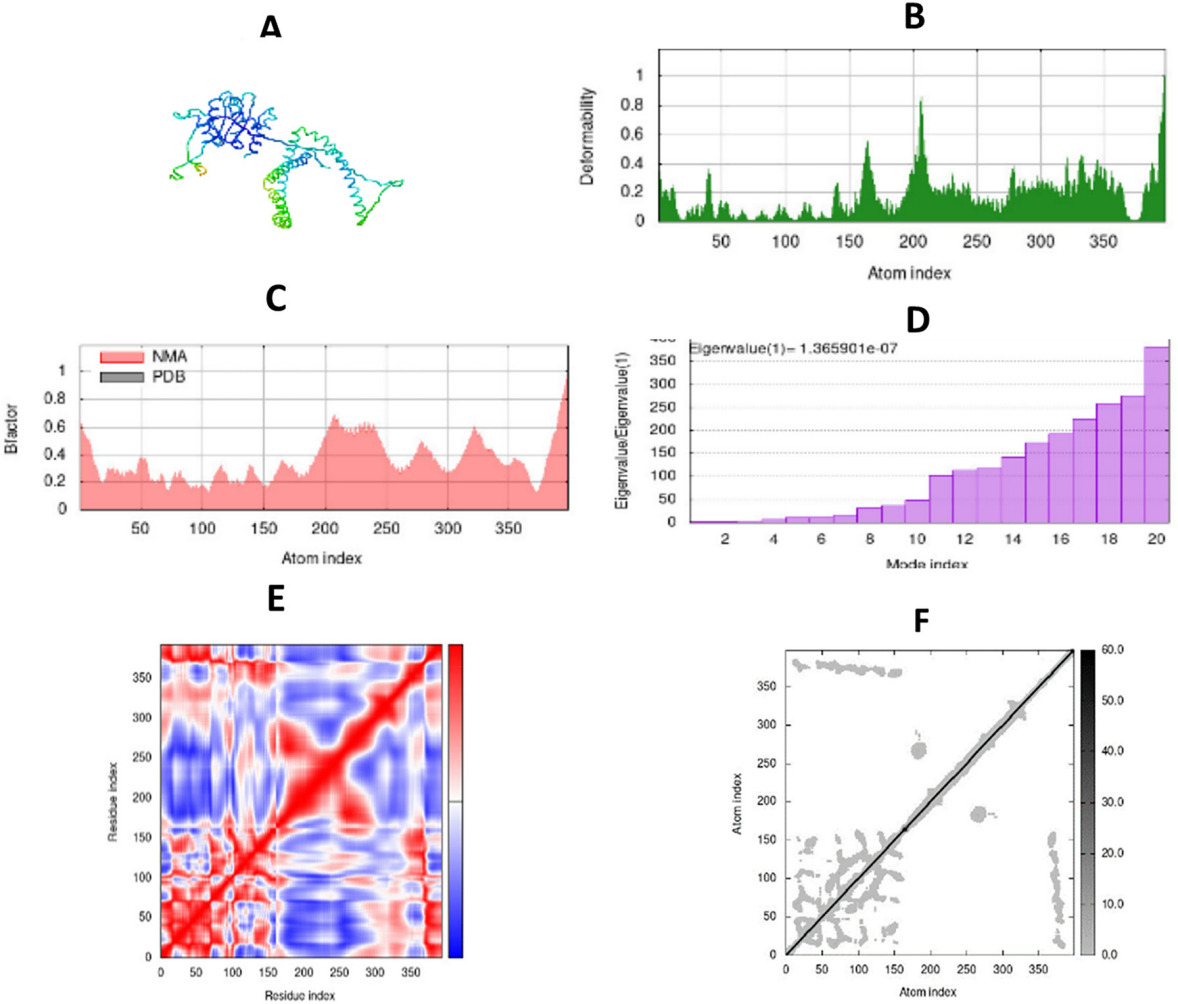
Molecular dynamic simulations of vaccine with TLR1. (A) dock complex of vaccine and TLR-1 generated by iMods, (B) deformability graph, (C) B-factor graph targeting NMA and PDB structure, (D) covariance map showing red color (correlated), blue (anti-correlated) and white (un-correlated, (E) elastic network represent stiffness of amino acid residues.

Vaccine 3D structure was predicted by Alphafold 2. Five different 3D structural models were provided by AlphaFold2, and the structure with the best prediction score was chosen for additional analysis. The Ramachandran plot analysis revealed that the majority of residues in the vaccine candidate were present within the most favored region. Additionally, the quality score of the top vaccine model is 96 %, indicating that refining the 3D structure is not mandatory as shown in Figure 7 (A-D). Furthermore, the ProSA-online server was used to assess the final vaccine 3D model’s quality which was further refined by the GalaxyWeb structure refining tool. The model quality was determined on the basis of Z-score value, the lower the Z-score value more competent is tertiary structure. The Z-score value of the selected model is −1.91 (Figure 7A–D).

## Molecular docking with TLR1 and TLR5

**Figure 8:**
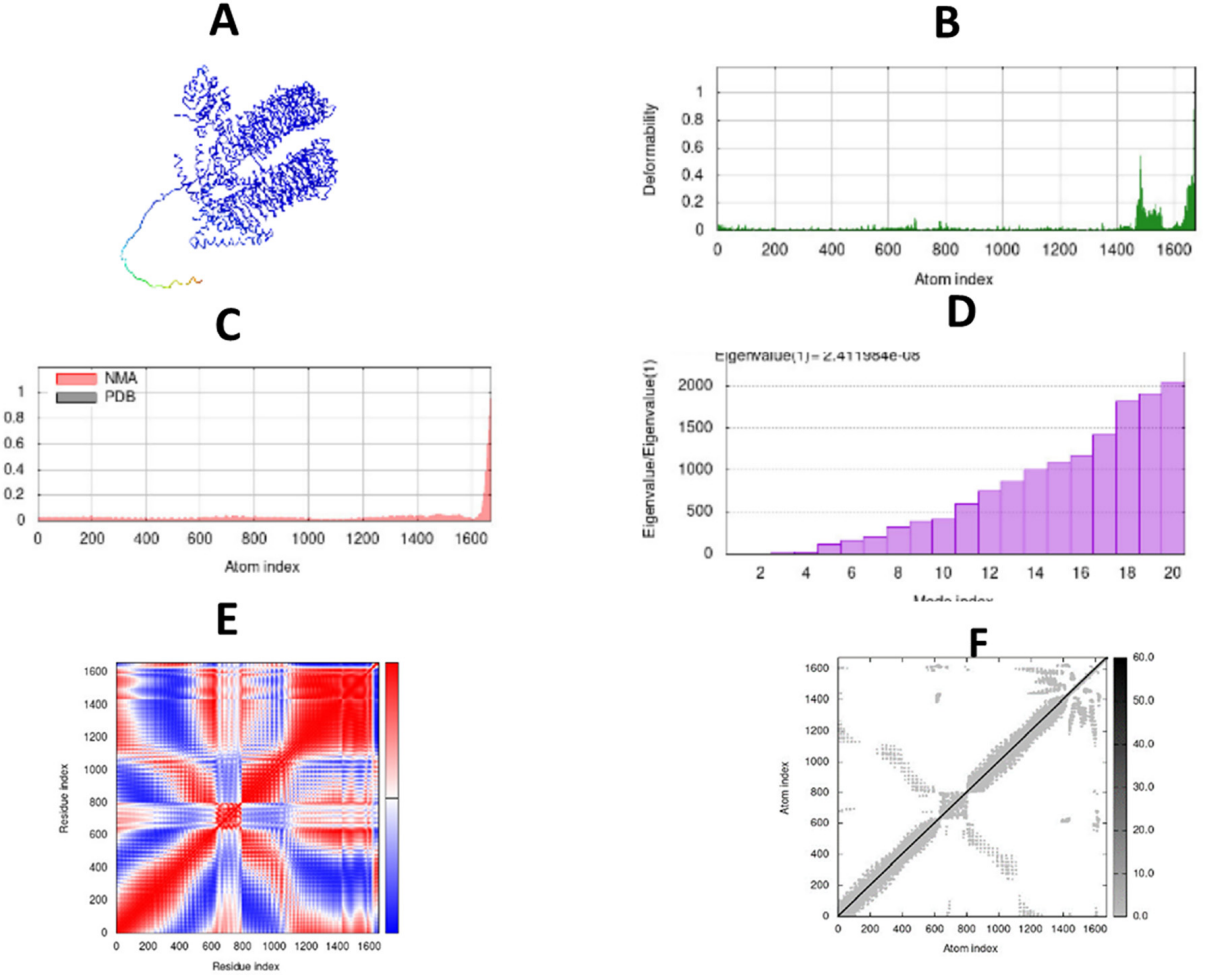
Molecular dynamic simulation of vaccine with TLR 5. (A) Dock complex of vaccine and TLR-5 generated by iMods, (B) deformability graph, (C) B-factor graph targeting NMA and PDB structure, (D) covariance map showing red color (correlated), blue (anti-correlated) and white (un-correlated, (E) elastic network represent stiffness of amino acid residues.

The developed vaccine component and the host’s innate and adaptive immune cells must interact with each other in order to activate cellular and humoral immunity. To determine its binding affinity, the vaccine was docked with the host immunological receptor. Molecular docking of vaccine was performed by using ClusPro v 2.0 with TLR1 (7NT7) and TLR5 (3J0A). For each docking, the ClusPro server produced 25 structures, in both cases, the model with the lowest binding affinity and the lowest intermolecular energy was chosen. The docking with TLR1 and TLR5 produced the lowest energy values, −1594.4kj/mol and −1628.2kj/mol, respectively (Figure 8A and B).

## Molecular simulations

The vaccine-TLR1 and vaccine-TLR5 complexes were employed for the molecular dynamic simulation study by using iMODS server. Peaks in the deformability graph show the amino acids that have coiled forms as well as the vaccine’s deformable loci. Protein flexibility is examined using a computer method called NMA (normal mode analysis). As shown in Figures 7 and 8, the complex’s connection between the PDB areas and Normal Mode Analysis is shown by the B-factor graph. The calculated Eigenvalue of TLR1 and TLR5 are 1.365901e – 07 and 2.411984e- 08, respectively. The eigenvalue value represents the energy that is required to deform the amino acid residues, the lower the eigenvalue, the easier it is to deform the molecule. The covariance matrix determines the coupling of amino acid residues regarding correlated and uncorrelated regions. Elastic networks represent results in the form of white and grey color, each dot in the graph showing stiffness. Molecules are stiffer and harder to deform presently in grey regions.

## MM/GBSA energy calculation

**Figure 9:**
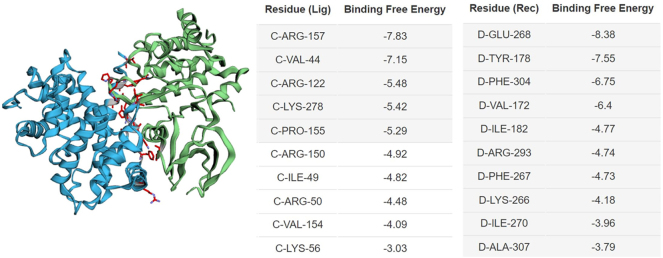
Free binding energy calculation of vaccine and TLR5 receptor protein complex.

The MM/GBSA calculation results from the Cluspro server revealed a notably favorable and strong binding interaction between TLR-5 (Immune Receptor) and vaccine candidate, with a free binding energy of −312.28 kcal/mol. This suggests the formation of a stable complex between them. Furthermore, the breakdown of individual energetic contributions is presented in Figure 9. Notably, the substantial Van-der Waals interactions at −13.65 kcal/mol underscore the favorable contacts between the molecules. Additionally, the presence of electrostatic forces at −6.2 kcal/mol indicates the participation of charged amino acids in the binding process. The comparison of different receptors based on their predicted binding free energies, as evidenced by ranking and scoring information, is valuable. TLR5 exhibits the most robust binding affinity, showing an MM/GBSA energy of −312.28 kcal/mol [30].

## Discussion

Streptococcus Intermedius is a β-hemolytic Gram-positive bacteria belonging to the family of *Streptococcus anginosus* group (SAG) also known as Streptococcus milleri [1]. At present, Streptococcus Intermedius is considered a virulent human pathogen causing multiple diseases like abscesses, endocarditis, and pneumonia without having any proper treatments [9]. Due to increased occurrence of this pathogen and antibiotic resistance, advanced techniques are required to overcome its pathogenicity [3]. Computational methods provide an effective approach to vaccine development. Nonetheless, conventional methods for developing vaccines are fraught with challenges such as time-consuming processes, exorbitant costs, and a higher likelihood of failure. An innovative and more streamlined strategy used by [10] for creating precise and stable vaccines is immunoinformatics. Additionally, these methods are also applicable for adjuvant identification to enhance immunological reactions of vaccines [4]. Prior research has effectively exploited computational-based approaches for vaccine designing against various pathogens. Such as [6] adopted different molecular modeling and other in silico approaches to determine the antigenic vaccine target of Streptococcus pneumonia. Their findings demonstrated the efficacy of using in-silico methods to select conserved peptide sequences as vaccine candidates. Likewise [8], employed bioinformatics tools for epitope prediction and analyzing their antigenicity and toxicity to construct a vaccine for *Mycobacterium tuberculosis*.

Selecting the appropriate protein antigen is a pivotal step in developing an epitope-based vaccine that is scientifically sound. In the present investigation, we harnessed the predictive power of two bioinformatic tools, namely Vaxijen and the AllerTOP server, to assess the antigenicity and allergenicity of the target protein to construct vaccine against Nipah virus [5]. Complementing this, we undertook a comprehensive pan genome analysis by extracting the proteome from a panel of bacterial species, encompassing 12 complete genome sequences [9]. The design of our chimeric multi-epitope vaccine entailed the identification of critical immunogenic elements, including B-cell epitopes and T-cell epitopes. Our approach incorporated 12 linear epitopes to stimulate B-cell responses, five cytotoxic T lymphocyte (CTL) epitopes targeting MHC class I (MHCI), and 5 T-helper lymphocyte (HTL) epitopes targeting MHC class II (MHCII). To ensure broad population coverage, the selection of HTL and CTL epitopes was informed by a thorough analysis of HLA diversity.

To contextualize our chimeric multi-epitope vaccine against *Streptococcus intermedius*, a comparison with existing vaccine candidates for related pathogens highlights its advantages. Unlike the pneumococcal polysaccharide vaccine (PPV23) for *Streptococcus pneumoniae*, which targets serotype-specific polysaccharides and offers limited cross-protection, our vaccine employs immunoinformatics to select 12 B-cell, 5 CTL, and 5 HTL epitopes with high antigenicity (VaxiJen >0.5), non-allergenicity (AllerTOP v2.0), and non-toxicity (ToxinPred), ensuring broader strain coverage and safety [6]. In contrast to *M. tuberculosis* vaccines focusing on single antigens like ESAT-6, our approach integrates multiple epitopes (IC50 <100 nM) predicted via NetMHCpan-4.1 and NetMHCIIpan-4.0, validated by molecular docking for stable receptor binding [10]. Compared to *Streptococcus pyogenes* M-protein-based vaccines, which face autoimmunity risks, our design minimizes such concerns through rigorous epitope filtering, offering a scalable, precise alternative, pending *in vivo* validation.

To demonstrate the stable connections between the vaccine and these immunological receptors, molecular docking and molecular dynamics simulations were performed [22]. These analyses highlighted the significant roles played by electrostatic and van der Waals energies in promoting effective binding between the vaccine and the receptors. During the molecular docking process, multiple hydrogen bonds were observed, and the subsequent molecular dynamics simulations demonstrated minimal variations [31]. These outcomes collectively confirm that the designed vaccine can effectively interact with immune receptors, thereby indicating the potential for this multi-epitope vaccination to provoke an effective immune response.

The research primarily relies on in-silico prediction, which has raised concerns about the potential limitations of supertype MHC epitopes in generating protective immunity rather than solely virus-reactive responses [28]. It is well-established that only a fraction of antibodies elicited by viral infections possess true neutralizing capabilities. In the absence of a comprehensive evaluation of the immune response induced by the proposed vaccine, it becomes challenging to assess its potential benefits for the human population. Consequently, additional *in vivo* studies are imperative to validate the efficiency and safety of the designed vaccine candidate.

## Conclusions

In conclusion, our research successfully developed a chimeric multi-epitope vaccine targeting the highly conserved epitopes of various *Streptococcus intermedius* serotypes. This innovation overcomes the limitations of current vaccines that are effective only against single serotypes. The new vaccine demonstrated high antigenicity, non-toxicity, and the ability to elicit robust immune responses across multiple serotypes, suggesting its practical application. Additionally, its structural stability in binding to human immune receptors ensures a durable and potent immune response. This development sets the stage for creating a chimeric multi-epitope vaccine targeting *S. intermedius* and establishes a strong foundation for designing epitope-based vaccines for other pathogens with multiple serotypes.
